# Enhanced school-based vision screening in Kumasi, Ghana: a pilot study to assess feasibility

**DOI:** 10.3389/fmed.2026.1911263

**Published:** 2026-08-17

**Authors:** Afua O. Asare, Enoch Ansah-Asiedu, Priscilla Ablordeppey, Emmanuel T. Doku, Gabriel K. Agbeshie, Seyram A. Gle, Nana Akwasi Owusu Mensah, Christiana Nyarko Yeboah, Ruby E. Adikah, Debora A. Baidoo, Christine K. Darko, Elisha E. Arkhurst, Melissa H. Watt, Sravanthi Vegunta, Eldrick A. Acquah, Hornametor Afake, Sylvia Agyekum, Kwadwo Owusu Akuffo

**Affiliations:** 1 Department of Ophthalmology & Visual Sciences, University of Utah, Salt Lake City, UT, United States; 2 Department of Population Health Sciences, University of Utah, Salt Lake City, UT, United States; 3 Department of Pediatrics, University of Utah, Salt Lake City, UT, United States; 4 Department of Optometry and Visual Science, College of Science, Kwame Nkrumah University of Science and Technology, Kumasi, Ghana; 5 University of Houston College of Optometry, Houston, TX, United States; 6 National Eye Care Unit, Ghana Health Service, Accra, Ghana

**Keywords:** child, global ophthalmology, health disparities, health literacy, health services accessibility, health services needs and demand, public health, referral and consultation

## Abstract

**Introduction:**

Childhood vision loss is a public health concern in Ghana, where access to eye care is limited. This prospective cohort pilot study was conducted to examine the feasibility of an enhanced, school-based vision screening protocol among children enrolled in a private and public school in Kumasi, Ghana.

**Methods:**

In November–December 2024, optometrists used an enhanced vision screening protocol to screen for eye disorders. Children were aged 4–22 in Primary 1 to Junior High School (JHS) 3 classrooms. Children identified with significant suspected eye disorders, as defined by established criteria, were referred off-site to an eye clinic for a comprehensive eye examination. Outcome measures included participation in vision screening, prevalence of suspected eye disorders, referral adherence, and program acceptability. Referral adherence was assessed using parent or guardian questionnaires, reports from referring optometrists, and interviewer-led surveys with children. Acceptability was assessed through focus groups with teachers, school administrators, and parents/guardians. Descriptive statistics were used to summarize participant characteristics and screening outcomes, and Pearson’s chi-square and two-sample *t* tests were used for bivariate comparisons by school type.

**Results:**

Out of 2,150 children attending both schools, 1,152 (53.6%) consented to being screened and completed screening. Complete data for 1,123 (97.5%) children was analyzed; 437 (38.9%) attended public and 686 (61.1%) private school. The average age of children screened was 10.2 (±2.6) years. The prevalence of suspected eye disorders was 34.0% (*n* = 382). Referral adherence was assessed for 299 children, of which 98 (32.8%) adhered to the referral. Teachers, school principals, and parents/guardians found the screening program acceptable.

**Conclusion:**

Half of the eligible children participated in the school-based vision screening program, a third were identified with untreated eye disorders, a third adhered to referral and the program was acceptable to key stakeholders. Enhanced school-based vision screening could be a feasible strategy to identify untreated eye disorders among school children in Kumasi, Ghana, however strategies to improve participation, and referral adherence are needed.

## Introduction

Childhood vision loss is a significant public health concern, particularly in low- and middle-income countries such as Ghana, where access to eye care is limited. About 90% of childhood vision loss in Ghana is considered avoidable (1), with the leading causes being uncorrected refractive error and amblyopia (2–4). Children with vision loss are often unaware of their condition and may not report symptoms, emphasizing the need for routine vision screening and follow-up comprehensive eye examinations (5–7).

Vision screening plays a critical role in identifying potential eye disorders in children (8, 9). In many high-income countries, vision screening is integrated into routine preventive health care for children through government-supported policies, which facilitate the early detection of eye disorders (10, 11). In Ghana, the School Eye Health Program provides free vision screening by optometrists and ophthalmic nurses in communities and schools with referrals to eye care clinics as needed (12). Despite its potential, the program is reported to be underutilized and inconsistently implemented (12).

The feasibility of school-based vision screening programs in Ghana has not been studied. Understanding feasibility is essential for implementing effective strategies to improve access to health care (13, 14). An enhanced school-based vision screening protocol (ESVSP) tailored to resource-limited settings was developed to bridge the gaps in access to pediatric vision care in Ghana. The ESVSP consisted of vision screening conducted by local trained and trainee optometrists, with referrals off-site for comprehensive eye exams based on defined criteria. This pilot study aims to examine the feasibility of the ESVSP among children enrolled in a private and public school in Kumasi, Ghana, by assessing participation in vision screening, prevalence of suspected eye disorders, referral adherence, and acceptability.

## Materials and methods

### Study design and sample

The cross-sectional pilot study was conducted in a public and a private school in Kumasi, Ghana. These schools were purposively selected because of their large student population [2,150 from Primary 1 to junior high school 3 (US grade 1–9 equivalent)] and proximity to each other [0.7 miles (on foot) and 1.7 miles (driving)] in the same neighborhood. Public schools in Ghana are operated by the Ghana Education Service (GES). Private schools, owned by private entities, offer either the GES-developed curriculum or international curricula such as the British Cambridge education system (15). Tuition is free in public schools through senior high school, in line with Ghana’s Free Compulsory Universal Basic Education (FCUBE) policy (15). Because of the free tuition in public compared to private schools in Ghana, a higher proportion of children from low-income families, as well as children working as domestic helpers in foster households (a common practice in Ghana) enroll (16).

Feasibility was assessed using implementation-relevant domains consistent with Bowen et al.’s framework (17). Specifically, we determined acceptability, demand/participation, and practicality, operationalized in this study through screening participation, referral adherence, and stakeholder acceptability. To assess acceptability and barriers to referral adherence, focus groups were conducted with teachers, school principals, and parents/guardians from each school.

### Ethical consideration

The study was conducted in accordance with the principles of the Declaration of Helsinki. The study proposal was reviewed and accepted by the Committee on Human Research Publication and Ethics (CHRPE/AP/866/24) at the Kwame Nkrumah University of Science and Technology, Kumasi. Approval to conduct the study was obtained from Heads of Schools, and the Head of the Eye Care Unit of the Ghana Health Service. During a Parent and Teachers’ Association meeting, research assistants explained the study procedures, risks, and benefits in English and Akan Twi. To participate, parents or guardians needed to sign consent forms. Parents or guardians reserved the right to exclude their children from the study at any time.

### Enhanced school-based vision screening protocol

The ESVSP was piloted at the public school from 4th to 8th November 2024, and at the private school from 25th November to 4th December 2024. Demographic data (e.g., sex, age) were collected from school enrollment records where available, as well as during in-person interviewer-led surveys with children. To maximize the return of consent forms, school administrators provided verbal reminders to parents/guardians during the consent period.

This study adopted a simplified, economical approach tailored to a low-resource setting. By aligning with the practical constraints and priorities of such environments, the protocol aims to enhance its relevance and appeal to national policymakers and decision-makers. Insights from this pilot implementation will serve as a foundation for strategic refinement of the program over time. The vision screening protocol was described as ‘enhanced’ because it was performed by local eye care professionals, namely 10 optometrists and four optometry students affiliated with the Department of Optometry and Visual Science at the Kwame Nkrumah University of Science and Technology. Also, the protocol included direct ophthalmoscopy as part of the vision screening. The ESVSP was reviewed with screeners prior to starting the study.

Distance visual acuity for the right, left, and both eyes was obtained with and without existing prescription using the Lea symbols in a well-illuminated room measuring 6 m (20 feet) distance. Near visual acuity was tested at the participants’ habitual reading distance. Pinhole examination was performed for participants with visual acuity of 20/40 or worse in children aged 4 years and 20/30 or worse in children older than 4 years. Refractive errors were estimated with a photoscreener (PlusoptiX S12C, software version 8.0.3.0; plusoptiX GmbH, Nuremberg, Germany) in a separate room with less illumination. A pen light was used to assess the external eye structures and anterior segment, while a direct ophthalmoscope was used to examine the optic nerve and macula of both eyes through an undilated pupil.

In this study, an eye disorder was defined as the presence of visually significant refractive error cases, and eye disorders identified in the screening using an ESVSP. In the absence of comprehensive eye exams, vision screenings do not assess overall eye health (18). Therefore, all cases identified in this pilot study were considered “suspected” and required a referral for further comprehensive examination and diagnosis.

Due to the lack of standardized national or international criteria, referrals from screening were made in line with recommendations from the American Academy of Pediatrics, the American Academy of Pediatric Ophthalmology and Strabismus (AAPOS), and the National Center for Children’s Vision and Eye Health (19–21). Referrals were made for children who were identified through photorefraction with suspected astigmatism of >1.75 D, myopia worse than <2.00 D, media opacities >0.1 mm, strabismus greater than 8 PD manifest, anisometropia >1.25 D, and hyperopia >4 D Children were also referred following visual acuity testing if they were 4 years old and unable to read the 20/40 line for either eye, or aged 5 and older and unable to read the 20/30 or 20/32 line for either eye, and had at least a two-line difference in visual acuities between the eyes. Children unable to complete any of the screening tests after two attempts were also referred. Because of the high prevalence of glaucoma in Ghana, even among children and young adults (22), children who had a suspicious disk defined as a Cup to Disk Ratio (CDR) ≥0.5, or a CDR difference of 0.2 between each eye, or the presence of a laminar dot sign were referred (23). The complete criteria for referrals are described in Supplementary Table S1.

Parents and guardians of children who did not pass the vision screening test were given a referral note summarizing the findings, along with the addresses and phone numbers of two optometry clinics within 3 miles (5 km) of the school to facilitate appointment scheduling. However, parents and guardians were informed that they could attend any eye clinic of their choice and were asked to bring the referral note to the appointment. Optometrists at the two provided referral clinics were asked to provide a report of the exam by completing a form on the back side of the referral note.

### Referral adherence

**Figure 1:**
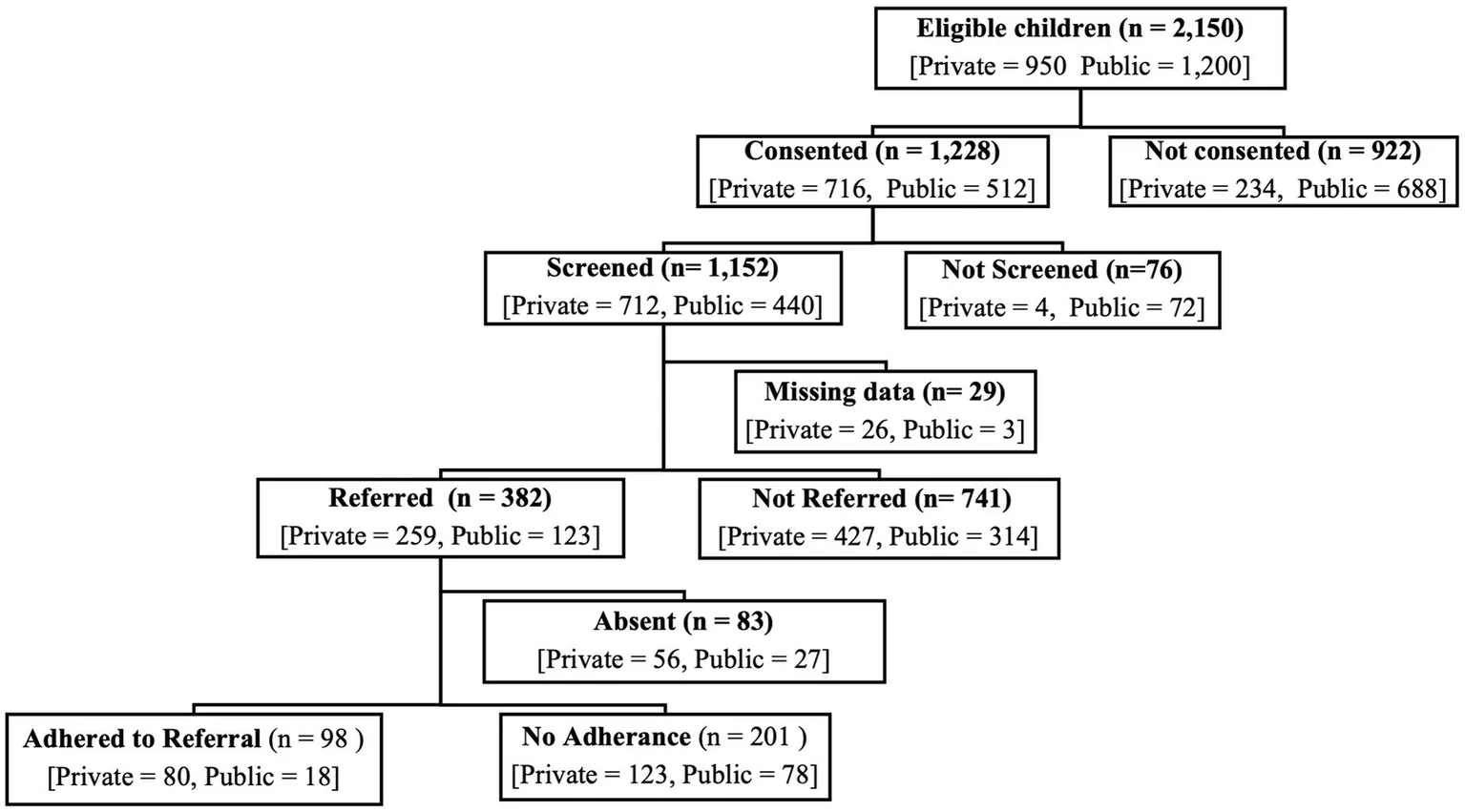
Flow chart representing the stages of GO-SCREEN pilot study.

To assess parents’ and caregivers’ natural response to referrals, the program did not cover the cost of the eye examination or prescription glasses. However, free comprehensive eye examinations were introduced later, after an initial period of observation. In June 2025, 6 months after vision screening, referral adherence was assessed using parent/guardian questionnaires, optometrist eye exam reports, and interviewer-led surveys with children (when parent/guardian questionnaires and optometrist reports were unavailable; Figure 1). Teachers and school principals assisted with collecting parent/guardian questionnaires and with sending reminders to parents/guardians regarding referral adherence.

From September 15th to October 4th, 9 months after the vision screening at the schools, free dilated comprehensive eye exams were offered to families at the KNUST Department of Optometry and Visual Sciences eye clinic located about 7.5 miles (12 km) from the schools. This special Saturday clinic was organized for children who had not adhered to the referrals 6 months after the vision screening and focus groups were completed. Prescription glasses were provided at no cost in November, following the comprehensive eye exams for the children who needed them.

### Acceptability of the enhanced school vision screening program

To determine stakeholder perspectives on the acceptability of the vision screening program and barriers to referral adherence, focus groups were conducted at each school with school administrators (teachers and school principals) as well as parents and guardians. Focus groups at the private school were conducted during parent-teacher conferences to maximize attendance.

### Data processing and analysis

Data from the ESVSP were collected and entered into a secure, HIPAA-compliant Research Electronic Data Capture (REDCap) (27, 28) tools hosted at University of Utah. Descriptive statistics were used to summarize participant characteristics and screening outcomes (participation in vision screening, prevalence of suspected eye disorders, and referral adherence). Bivariate comparisons by school type were conducted using Pearson’s chi-square tests for categorical variables. Age was compared between school types using a two-sample t-test for continuous data.

Discussions from focus groups were audio-recorded, transcribed, and analyzed using deductive coding and thematic analysis. To enhance rigor, five independent coders coded transcripts; discrepancies were resolved by consensus, and findings were triangulated across participant groups.

## Results

### Participation in vision screening and participant demographics

Of the 2,150 schoolchildren attending the two schools, 1,228 (57.1%) had a parent or guardian’s consent to participate in screening. Of the consented children, 76 (6.2%) were absent on the screening days, and 1,152 (93.8%) were present and screened for eye disorders. The overall participation rate in vision screening was 53.6%; 75.0% in the private school and 36.7% in the public school (Table 1). 648 (57.7%) children screened were female, of which 266 (60.9%) were enrolled in public school. The average age of children screened was 10.24 (±2.60) years, 10.90 (±2.61) years in public and 9.83 (±2.51) years in private school (Table 2). Twenty-nine children were excluded from the sample due to missing data. Therefore, data for 1,123 enrolled students were included for analysis.

**Table 1:** The number and proportion of children who received consent forms, and were screened by type of school.

School type	Student population	Consented	Screened
Public	1,200	512 (42.67)	440 (36.67)
Private	950	716 (75.37)	712 (74.95)
Total	2,150	1,228 (57.12)	1,152 (53.58)

Proportions represent row percentages.

**Table 2:** Descriptive characteristics of the number and proportion of children screened in the vision screening pilot study by type of school.

Characteristic	Total (*n* = 1,123)^*^	Type of school		*p*-value
		Public (*n* = 437)	Private (*n* = 686)	
Sex				0.09
Female	648 (57.70)	266 (60.87)	382 (55.69)	
Male	475 (42.30)	171 (39.13)	304 (44.31)	
Age, years^**^	10.24 (2.60)	10.90 (2.61)	9.83 (2.51)	0.00

Proportions represent column percentages.

^*^29 children were excluded from the total number of children screened because of missing data.

^**^Age is represented by the mean (±standard deviation) for *n* = 1,115. 8 children were excluded because of missing data.

### Prevalence of suspected eye disorders warranting referral

**Figure 2:**
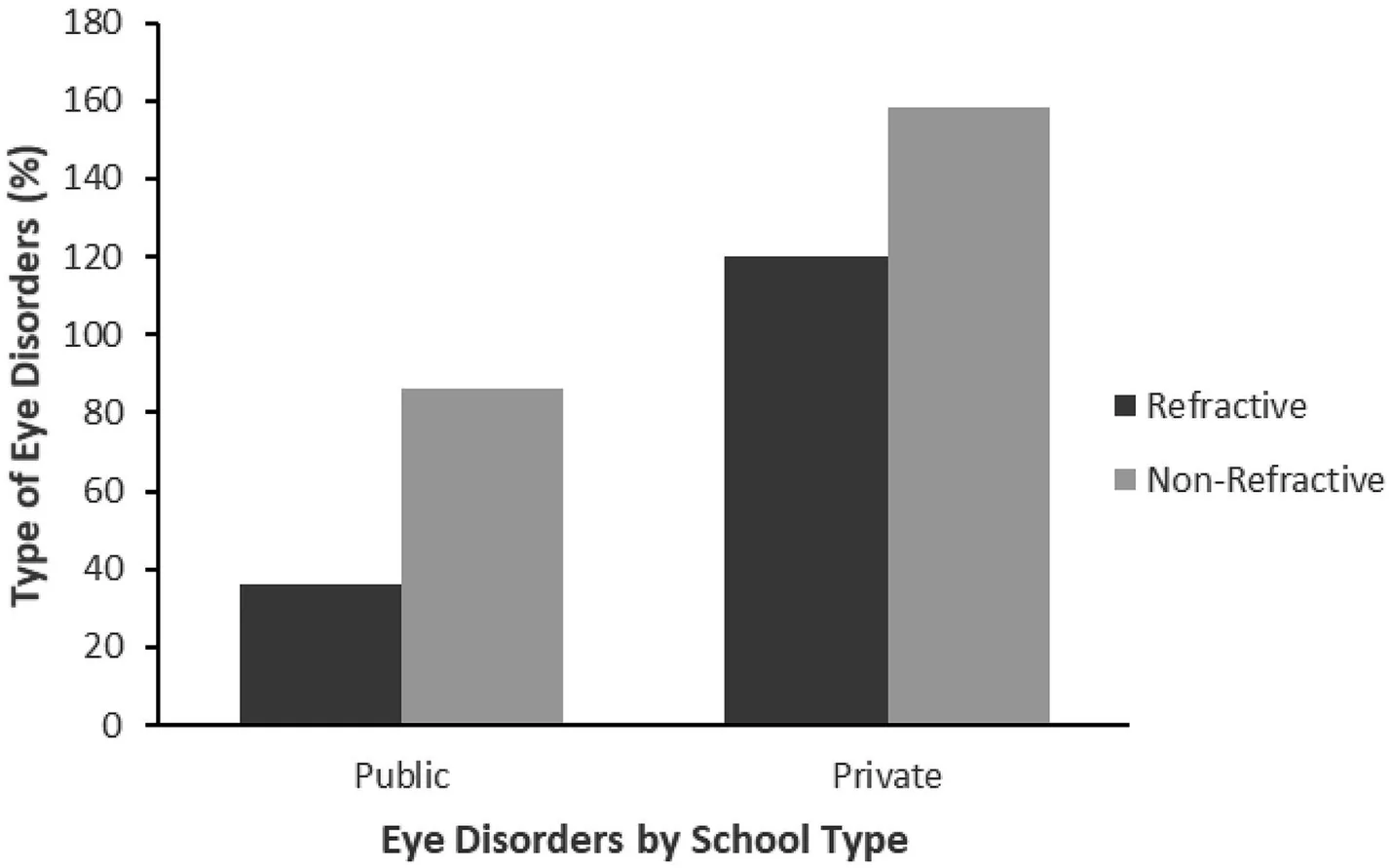
Bar graph reporting the proportion of children referred by type of eye disorder and school following vision screening.

Of the 1,123 children screened, 382 were identified with an eye disorder, representing an overall prevalence of 34.0%. All 382 children were referred to an eye clinic for a comprehensive eye exam by an eye care professional. The majority of children referred [244/382 (63.9%)] were identified with non-refractive eye disorders, while 156 (40.8%) were identified with refractive disorders (Figure 2). Children were counted in both groups if they had both non-refractive and refractive eye disorders. For those with non-refractive eye disorders, posterior segment anomalies [210/382 (55.0%)] were the most predominant, particularly suspicious disks or laminar dot sign [194/210 (92.4%)]. For children with refractive disorders, astigmatism [82/156 (52.6%)] was the common subtype, followed by myopia [26/156 (16.7%)].

### Referral adherence

Of the 382 children referred, adherence data were collected for 299 (78.3%) children 6 months after vision screening. Eighty-two children no longer attended the school, and one child was excluded because of a data entry error. Of the 299 children for whom data were available, 98 (32.8%) adhered to the referral; 18 (18.4%) in the public school, and 80 (81.6%) in the private school. 14 optometrist reports were retrieved from the referral clinics, suggesting that most children either attended other optometry clinics besides the two provided by the research program or forgot to bring the referral report to the appointment.

### Acceptability of the vision screening program

A total of seven focus groups were conducted with about six participants per group. The results of focus groups with school administrators and parents/guardians indicated that the vision screening program was acceptable to both groups, and there was interest in having the program provided regularly (at least once a year) at each school. Parents/guardians most appreciated the convenience of in-school screening. Suggestions were made to address communication gaps, such as using established school electronic communication channels like WhatsApp for consent, providing verbal explanations of results, and providing financial assistance.

## Discussion

In this pilot study, an ESVSP was assessed for feasibility. Participation was moderate, referral adherence was low, and stakeholder acceptability was high. About half of the eligible children participated; one-third of those screened had suspected eye disorders, and one-third of the referred children adhered. These findings highlight both the potential of school-based vision screening to identify unmet eye care needs and the implementation challenges that must be addressed to improve participation and referral adherence.

Higher participation rates in school vision screening have been reported in Ghana (2, 24–26). Kumah et al. reported a participation rate of 91.2% among public school children aged 6–16 years during a screening initiative in the Kwabre East District of the Ashanti region (26). A separate study by the same lead author reported 99.2% participation among private school children aged 12–15 years in Kumasi (2). The lower participation in the present study, may reflect differences in how children were recruited, institutional support from school administrators, parent/guardian enthusiasm, and how consent was obtained. However, limited detail on whether consent was opt-in or opt-out in the earlier studies makes direct comparison with the present study difficult.

Global rates of participation in school-based vision screening vary, with higher participation observed in programs integrated into school health systems and programs that use opt-out consent models. For instance, programs offering opt-out models, participation rates of 97% were recorded in Bradford, United Kingdom (29), and 89% in Ontario, Canada, while only 62% using an opt-in consent model (30). In India and other lower-middle-income countries, school-based vision screening programs integrated into existing school health systems reported higher participation rates (31, 32). Participation in this study was high in the private school, similar to reports by Kumah et al. (2). The lower participation in the public school may reflect lower parental health literacy and weaker institutional support for vision screening than in the private school (33, 34), suggesting that social determinants of health may influence participation in school vision screening.

Referral adherence following school-based vision screening remains a persistent challenge. In this study, 33% of referred children adhered to the referral. This estimate is consistent with prior studies reporting adherence rates ranging from 30 to 60%, and lower rates in medically underserved communities (35–37). These findings underscore the need for integrated referral systems and community-based support to improve continuity of care. Factors contributing to poor adherence include limited access to eye care providers, low parental awareness, and socioeconomic barriers (38–40). The lower referral adherence observed in the public school may reflect socioeconomic differences, lower parental awareness of eye health needs, weaker school engagement, and greater barriers to accessing follow-up care. However, because these factors were not directly measured, this interpretation should be viewed as exploratory.

Participation in school vision screening and adherence to post-screening referrals may be improved by simplifying consent procedures, using electronic or school-based reminder systems, engaging teachers and school administrators as active partners, and reducing financial and logistical barriers to follow-up care. In this study, stakeholders also suggested using WhatsApp and providing verbal explanations to improve communication, as well as providing financial assistance to families. A model that has been often used is mobile outreach clinics for comprehensive dilated eye exams in school settings for children who require referrals after vision screening (41, 42). However, this model requires substantial start-up resources and may not be cost-effective in all settings.

The prevalence of eye disorders among screened students was 34%. Other studies in Ghana and other West African countries have reported prevalence rates ranging from 10.1 to 52.5% (3, 43–46). These variations in prevalence may be attributed to differences in sampling methods, study designs, diagnostic criteria, geographic locations, and participant age ranges. Given the heterogeneity in study protocols across the literature, caution should be exercised in generalizing findings from any single study to broader populations.

Study findings highlight school age as a critical window for the early identification of glaucoma suspects through enhanced screening, particularly given the risk of irreversible visual impairment associated with delayed diagnosis. The substantial burden of posterior segment anomalies supports the implementation of routine pediatric eye screenings as a strategy for early intervention and management. Suspicious disks were the most common eye disorder observed, followed by refractive error. This finding contrasts with other studies that reported allergic conjunctivitis (44) or refractive error (3, 45, 47) as the most common eye disorder. Such differences may be attributed to variations in participants’ ages, geographic locations, diagnostic criteria, refractive error thresholds, and the season during which the studies were conducted. The prevalence of glaucoma in Ghana is high, even among children and young adults, and its early stages are often asymptomatic (22, 48–50). However, direct ophthalmoscopy through an undilated pupil has limited ability to diagnose glaucoma or optic neuropathy, leading to false positives. Also, suspicious disks with an abnormal cup-disk ratio may be physiological (51). For this reason, posterior segment findings should not be interpreted as confirmed pathology. Nonetheless, these cases still warrant a comprehensive eye examination by an eye care professional (52).

To prevent amblyopia, early detection and timely correction of risk factors, such as significant refractive errors and anisometropia, are essential. Refractive error was the second most common suspected eye disorder among children referred for a comprehensive eye exam. Specifically, astigmatism was the most common suspected refractive error subtype. Normal postnatal eye development generally progresses from moderate hyperopia at birth toward emmetropia or low hyperopia at school age, with astigmatism typically decreasing with age (53, 54). However, environmental and behavioral risk factors, such as increased near work and reduced time spent outdoors, which are common among school-aged children, may predispose them to the later development of myopia and/or astigmatism (55, 56). More children in the private than in the public school had suspected myopia. This result may reflect increased near work and/or reduced outdoor time among children in private schools. Public health strategies that encourage increased outdoor activities and classroom designs that enhance natural lighting may be beneficial because they can help protect against myopia (57–59).

The ESVSP was acceptable to teachers, school principals, and parents/guardians who participated in focus groups. Participants described the program as convenient and expressed a desire for vision screening to be integrated into the school health system and provided at regular intervals. However, they reported gaps in communication regarding program procedures and the pathways to obtaining glasses or specialty care. Previous studies have reported similar enthusiasm for school-based vision screening programs, as well as communication gaps (60–63). A subsequent qualitative analysis will explore stakeholder perspectives on barriers to participation and referral adherence in greater depth.

A major strength of this study is its assessment of the feasibility of an ESVSP among school-aged children. Unlike previous studies conducted in Ghana (44, 46), this study includes children from both private and public schools. Additionally, we adopted a vision screening protocol with off-site referrals to minimize costs and increase its appeal to national policy and decision makers, while allowing us to evaluate its impact on participation rates. Insights gained from this initial implementation can inform strategic refinements to the program over time.

This study has some notable limitations. Because vision screening was conducted outside a clinic, cycloplegic refraction, the gold standard for assessing refractive error, was not performed. Therefore, the refractive errors reported are estimates derived from photoscreening. Although photoscreeners are well-suited for mass school-based screening because they are easy to use, have rapid measurement times, and have relatively high sensitivity and specificity, non-cycloplegic photorefraction may underestimate the spherical component of refractive error (44, 46). Another limitation is that the schools were purposively, rather than randomly, selected, which limits generalizability. The two schools were chosen to reflect typical differences in socioeconomic composition between private and public schools in Kumasi, while minimizing confounding related to proximity to specialty eye care. Accordingly, the primary aim of the study was not to generalize to all school-aged children in Ghana by making population-level inferences, but to assess feasibility. In addition, despite school administrators providing verbal reminders, the overall participation rate was 53.6%. Most non-participation was due to failure to return parental consent forms in this opt-in design. However, participation bias could not be determined because data were not collected from non-participants. Similarly, bias related to referral adherence could not be assessed because data on referral adherence were available for 78% of referred children, and absent for the remaining children, largely because some were no longer enrolled at the school. For this reason, the adherence estimate should be interpreted cautiously.

## Conclusion

To conclude, findings from the pilot study demonstrate that school-based vision screening could be a feasible public health intervention and essential to identify the high rates of eye disorders among school-aged children. However, efforts are needed to improve participation and referral adherence, especially in public schools. Future research will investigate the barriers to participation and referral adherence, as well as strategies to address them. Study results will be essential to developing a strategic vision screening intervention to improve the effectiveness and equity of pediatric eye care services in school-based settings in Ghana.

## Data Availability

The datasets presented in this study can be found in online repositories. The names of the repository/repositories and accession number(s) can be found at: 10.17605/OSF.IO/UH8PX.
