# Supplementary material for: Enhanced school-based vision screening in Kumasi, Ghana: a pilot study to assess feasibility
Source: Front Med (Lausanne). 2026 Aug 17;13:1911263. doi: 10.3389/fmed.2026.1911263 (PMC13526608; doi:10.3389/fmed.2026.1911263)
Supplement: Supplementary file 1 [file Table_1.DOCX]

Supplementary Materials

S1 Table. Age-based referral criteria used for GO-SCREEN

| ARF or Refractive Error | Age | Threshold |
| --- | --- | --- |
| ARF (severity ranked) |  |  |
| Media opacity | All ages | > 0.1mm |
| Strabismus | All ages | > 8 PD manifest |
| Anisometropia | All ages | > 1.25 D |
| Hyperopia | All ages | > 4.00 D |
| Visually Significant Refractive Errors |  |  |
| Astigmatism | ≥ 4 years | > 1.75 D |
| Myopia | ≥ 4 years | < -2.00D |

Abbreviations: ARF, amblyopia risk factor; D, diopters; PD, prism diopters. Note. American Academy of Pediatric Ophthalmology and Strabismus (AAPOS) uniform guidelines for instrument-based pediatric vision screen validation 2021 by Arnold et al.^1^

a) Age-dependent critical line for visual acuity testing

| Age (months) | Visual Acuity Critical Line to Pass |
| --- | --- |
| 48-59 | 20/40 |
| ≥60 | 20/30 (or 20/32 line on some charts) |

Note. “Procedures for the Evaluation of the Visual System by Pediatricians” 2016 by Donahue et al. ^2^

**Children who could not complete any of the screening tests after two screening attempts or with a two-line difference in visual acuities between the eyes were also referred.**
